# Effects of Atmospheric Aging on the Respiratory Toxicity of Polystyrene Nanoplastic Particles

**DOI:** 10.1021/acs.chemrestox.5c00237

**Published:** 2025-11-03

**Authors:** Alana J. Dodero, Olivia C. G. Lampe, Sahir Gagan, Sining Niu, Natalie M. Johnson, Yue Zhang

**Affiliations:** † Department of Atmospheric Sciences, 14736 Texas A&M University, College Station, Texas 77843, United States; ‡ Department of Environmental and Occupational Health, Texas A&M University, College Station, Texas 77843, United States; § Interdisciplinary Faculty of Toxicology, Texas A&M University, College Station, Texas 77843, United States

## Abstract

Inhalation exposure to nanoplastic particles (NPPs) can lead to significant pulmonary toxicity; however, the effects of environmental processing on their toxicity remain poorly understood. This study examines the toxicity of polystyrene (PS) NPPs on lung cells following controlled atmospheric aging. Human bronchial epithelial cells (16HBE) were cultured in vitro at the air–liquid interface and acutely exposed to oxidized PS NPPs through electrostatic precipitation. Expression of proinflammatory genes interleukin-8 (*IL*-8) and tumor necrosis factor alpha (*TNF*-α) was significantly elevated at 6 and 48 h postexposure to aged NPPs, with corresponding increases in interleukin-6 (IL-6) protein levels supporting an inflammatory response. The oxidative stress marker heme oxygenase-1 (HO-1) also showed significantly increased expression at 6 h postexposure, supported by protein analysis. Atomic force microscopy (AFM) and aerosol mass spectrometry (AMS) revealed increased surface roughness and oxygen to carbon ratios in the atmospherically aged NPPs. Together, these results demonstrate that atmospheric aging alters the chemical composition and surface morphology of PS NPPs, enhancing proinflammatory and oxidative stress responses in bronchial epithelial cells, highlighting the critical role of environmental processing in determining the toxicity of nanoplastics.

## Graphical abstract

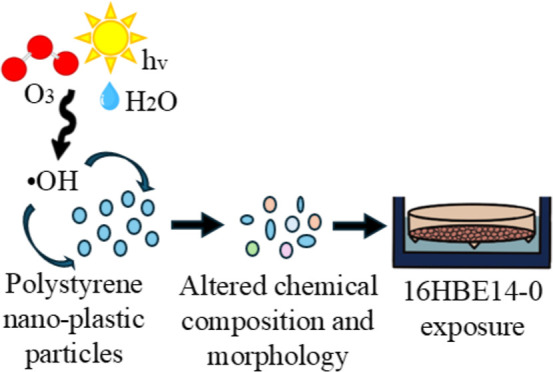

## 1 Introduction

Plastic pollution is widespread, contaminating diverse ecosystems worldwide.^1–3^ By 2060, global plastic waste generation is projected to exceed 1 billion tons,^4^ with less than 9% being recycled^5–7^ and 12% being incinerated,^7^ leaving the majority to accumulate in the environment.^1^ The routine production and disposal of plastic materials lead to the widespread emergence of micro- and nanoplastic particles (MNPPs).^1–3,8–11^ Microplastics are typically definded as particles of 1–5000 μm, while nanoplastics are less than 1 μm. They are produced from either primary or secondary sources.^12–14^ Manufacturers intentionally produce primary MNPPs for applications such as personal care and cleaning products,^6,15,16^ while secondary MNPPs form from the degradation of macro-plastics and contribute to a large percentage of global plastic pollution.^16^

MNPPs are emitted throughout their production and use,^17^ making them prevalent in terrestrial, aquatic, and atmospheric ecosystems^1,18–23^ worldwide.^24–27^ Due to their small size, nanoplastic particles (NPPs) can remain airborne for hours to days,^23,28^ facilitating long-range atmospheric transport from emission sources to remote regions.^29^ Their ubiquity, small size, and ability to penetrate biological tissues^30^ raise significant health concerns. NPPs can enter the human body through inhalation, ingestion, and dermal contact,^18,31^ and bioaccumulate in organs.^8,32^ Once inhaled, NPPs can penetrate deeply into the lungs and alveoli^33–35^ and cross physiological barriers, including the blood–brain barrier,^36,37^ where they may trigger inflammatory responses and oxidative stress.^38–41^

The toxicity of atmospheric NPPs is determined by their physical characteristics, chemical composition, environmental interactions, and degree of aging.^6^ Among environmental aging processes, photooxidation–particularly the reaction of NPPs with hydroxyl radicals (^•^OH)–plays a critical role in altering their physiochemical characteristics while they are suspended in the air.^42,43^ Photooxidation reactions introduce functional groups,^44^ modify surface morphology, and promote structural degradation,^45^ potentially altering cellular interactions and amplifying adverse health effects. Despite evidence that such transformations are likely during atmospheric suspension, the toxicological implications of NPP aging remain poorly understood.

While most studies on MNPP toxicity focus on the gut microbiome and liver, evidence suggests that NPPs can induce cytotoxicity in a dose-dependent manner. Polystyrene (PS) NPPs between 20 and 560 nm are absorbed internally by various cell lines,^46–48^ with toxicity likely mediated by reactive oxygen species (ROS)-driven inflammation and membrane damage.^49^ Clearance of microplastics relies heavily on mechanical methods such as trapping particles in the mucus lining of the lungs, phagocytosis via alveolar macrophages, and transport through lymph to the thoracic lymph nodes of the lung before emptying into systemic circulation.^50^ MNPPs such as polyvinyl chloride (PVC) have been shown in vitro to induce cytotoxicity and upregulate inflammatory cytokines such as interleukin-6 and −8 (*IL*-6 and *IL*-8), and tumor necrosis factor alpha­(*TNF*-α) in A549 cells and THP-1 cells.^51^

Traditional in vitro studies of the respiratory toxicity of NPPs primarily relied on submerged cell culture models using pristine, spherical PS particles.^52^ While these studies provide valuable baseline information, they neglect two critical aspects of real-world exposure: (1) atmospheric oxidative aging that alters particle properties before inhalation, and (2) direct particle deposition onto airway epithelia. The air liquid interface (ALI) exposure model addresses these gaps by mimicking the lung environment and enabling direct delivery of airborne particles onto epithelial cells without interference from culture media.^53^ This system allows for more physiologically relevant assessments of particle-induced responses. Fewer studies have utilized the air liquid interface (ALI) cell culture model; however, studies employing the ALI cell culture model suggest that PS NPPs do not induce inflammation or affect cell viability in air liquid interface cultures of A549 and BEAS-2B cells.^54^ The ALI cell culture model allows direct deposition of airborne particles onto cells, minimizing interactions with cell culture media and enabling accurate dose calculations. In the ALI system, particles and vapors are directly deposited onto the cells by removing the apical layer of media from the transwell insert.^53^ Removing the apical layer of the cell culture media eliminates the risk of physiochemical changes between the NPPs and the media, with added benefits of simpler and more accurate calculations when deriving the delivered dose.

The objective of this study was, therefore, to mechanistically investigate how photooxidation influences the toxicity of PS NPPs. PS NPPs with 500 nanometer diameter were atomized and exposed to ^•^OH, ozone, and UV–C radiation (λ = 254 nm) in a Potential Aerosol Mass (PAM) oxidation flow reactor to simulate 0, 9.1 ± 1.1, 16.5 ± 2.3, 20.9 ± 1.1 days of atmospheric aging. Human bronchial epithelial cells (16HBEs) were then exposed to 6.25 μg/mL of PS NPPs using a CelTox Sampler for 3 h, with cellular responses assessed both 6 and 48 h postexposure. By integrating controlled oxidative aging with physiologically relevant exposures, this study provides new mechanistic insight into how atmospheric processing alters the cellular responses of NPPs.

## 2 Experimental Procedures

### 2.1 Polystyrene Solution Preparation and Dosage

Monodisperse PS NPPs of 500 nm diameter (Sigma-Aldrich Inc.) were diluted from an aqueous suspension to a concentration of 0.3% (w/w) using ultrapure water. The PS NPP solution was atomized using a constant output atomizer (Model 3076, TSI Inc.) to generate aerosolized PS particles. Atomized PS NPPs were passed through an in-house silica diffusion dryer to remove excess water and then sampled by a Scanning Electrical Mobility Spectrometer (SEMS) (Model 2100, Brechtel Inc.) to determine the number concentration and size distribution. PS NPPs passed through the CelTox sampler (MedTec Biolab, Inc.) while enhancing the particle deposition by applying an electric charge to the particles and using electrostatic deposition. The concentration of PS aerosols was also measured at the exhaust of the CelTox sampler to determine the concentration of PS aerosols being deposited inside the CelTox sampler. The collection efficiency of the system was determined by comparing the PS NPP concentration before and after the CelTox sampler. Figure S1 of the Supporting Information Section S1 illustrates the differences in PS aerosol concentration before and after being deposited in the CelTox sampler. The dosage of PS aerosols to the cells was then determined by (eq 1).

1 $$D=\frac{C\times f\times t\times ce}{SA}$$

here, *D* represents the dosage, *f* represents the flow rate going into the CelTox sampler, *t* represents the total collection time, ce is the collection efficiency, and SA is the surface area of the electrostatic deposition flow path. During each experiment, PS aerosols entered the CelTox at a flow rate of 2.2 L per minute (lpm), and the cells were exposed for 180 min. Therefore, the total exposure of the cells for each experiment was 1.5 $\frac{\mu g}{cm^{2}}$ (or 6.25 $\frac{\mu g}{mL}$ as determined by the volume and surface area of the CelTox sampler inserts). A single exposure concentration of 6.25 $\frac{\mu g}{mL}$ of polystyrene nanoplastics was used as the focus of this study to assess the differences in cellular responses between oxidation states, rather than to establish a dose–response relationship. The exposure duration was set at 3 h to allow all treatment groups to be completed within one circadian cycle, with each exposure paired to a filtered air control to reduce time-dependent variability. The selected dosage additionally falls within values reported in literature.^4^

### 2.2 Oxidation of Polystyrene Nanoplastics

This study included two oxidation conditions: (1) ozonolysis of PS NPPs, and (2) oxidation of PS NPPs against ^•^OH in the presence of ozone and varying UV fluxes. Experiments were conducted by increasing the flux of UV–C radiation to elevate the ^•^OH concentration, representing PS NPP photochemical aging in the atmosphere for various periods. In the case of ozonolysis, Gagan et al.^55^ showed that polystyrene ozonolysis was negligible under similar experimental conditions. For ^•^OH-driven oxidation, Gagan et al. hypothesized that reactions occur primarily through H-abstraction and ^•^OH addition, leading to the formation of oxygenated functional groups.

To perform the oxidation experiments for PS NPPs, atomized PS NPPs first passed through a silica diffusion dryer at a flow rate of 3.5 lpm to remove excess water. The atomized particles then entered the Potential Aerosol Mass-Oxidation Flow Reactor (PAM-OFR) for aging via photo-oxidation. Throughout the experiments, ^•^OH was generated inside the PAM-OFR by the photolysis of ozone (O_3_) with UV–C (λ = 254 nm) to produce singlet oxygen (eq 2), which subsequently reacted with water vapor to form ^•^OH (eq 3).

2 O3+hv→O2+O(D1)

3 O(D1)+H2O→2OH·

O_3_ was generated by irradiating zero air with a mercury lamp (185 nm) and entered the PAM-OFR at a flow rate of 2 lpm. The RH was controlled by passing 2 lpm of zero air through a humidified Nafion tube (Perma Pure LLC, Model PD-07018T-12MSS) and into the PAM-OFR. PS aerosols were exposed to O_3_, UV–C, and ^•^OH at a relative humidity (RH) of 56 ± 5%, representative of typical ambient daytime conditions.^56–58^ The water vapor content inside the PAM-OFR was maintained at 1.7 ± 0.2%, calculated using eq 4.

4 $$wv=\frac{RH^{*}e_{s}}{p_{stp}}$$

Here *e*_s_ is the saturation vapor pressure inside the PAM-OFR, and *p*_stp_ is 1013 hPa. The residence time inside the PAM-OFR was 106.4 s (eq 5), based on the PAM-OFR volume of 13.3 L and total flow rate of 7.5 lpm.

5 $$t=\frac{V}{f}\times 60$$

After passing through the PAM-OFR, the PS aerosols went into an ozone denuder to remove the ozone from the aerosols. Then, the oxidized aerosols entered a SEMS, high-resolution time-of-flight aerosol mass spectrometer (HR-ToF-AMS, Aerodyne Research Inc.), and the CelTox Sampler. The concentration of PS NPPs was monitored with the SEMS before entering the CelTox sampler throughout the experiments to ensure the stability of the PS aerosol concentration. A detailed schematic of the experimental setup is shown in Figure 1.

**Figure 1:**
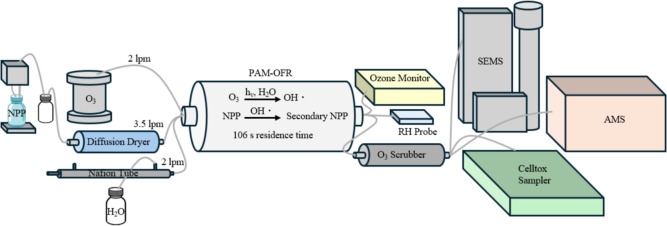
A schematic diagram to show the experimental setup and instrumentation for PS NPP generation, photo-oxidation, size characterization, and toxicity evaluation.

The ^•^OH exposures used in this work follow the methodology of previous studies.^59,60^ In short, ^•^OH exposures and UV photon flux rates were estimated using the PAM chem model program.^61^ During the different experiments, the voltage of the UV–C lights was changed, while the voltage of the ozone lamp remained constant. The model used O_3_ concentration, residence time of the PAM-OFR, water vapor content, and temperature inside the PAM-OFR to determine the UV-flux for each experiment as discussed previously.^59–61^ The derived UV-flux was then used to estimate the ^•^OH exposure for each oxidation condition. The flux of UV–C varied from 0–1.9 × 10^15^ ± 3.8 × 10^14^ cm^–2^ s^–1^, leading to ^•^OH exposures from 0–2.7 × 10^12^ ± 2.9 × 10^11^ molecules s cm^–3^, or equivalently, 0–(20.9 ± 1.1) days of aging in the atmosphere. The equivalent number of days for each condition was calculated by dividing the calculated ^•^OH exposure by the ambient ^•^OH concentration by assuming an ambient ^•^OH concentration of 1.5 × 10^6^ cm^–3^.^59,60^ Four different experiments were done by varying UV–C flux, and three repetitions were completed for each experiment. The conditions for each experiment are summarized in Table 1.

**Table 1:** Experimental Conditions for Oxidation Experiments

number concentration (number cm^–3^)	mass concentration (μg m^–3^)	O_3_ concentration (ppm)	^•^OH exposure (molecules s cm^–3^)	equivalent photochemical aging (days)	C_6_H_6_^+^ concentration (μg cm^–3^)	C_8_H_8_^+^ concentration (μg cm^–3^)	O/C ratio
(3.12 ± 0.03) × 10^4^	(1.10 ± 0.02) × 10^3^	4.46 ± 0.20	(2.71 ± 0.29) × 10^12^	20.9 ± 1.1	4.57 ± 0.17	4.41 ± 0.14	(1.21 ± 0.37) × 10^–2^
(3.11 ± 0.04) × 10^4^	(1.11 ± 0.02) × 10^3^	4.68 ± 0.26	(2.14 ± 0.68) × 10^12^	16.5 ± 2.3	4.56 ± 0.26	4.39 ± 0.25	(1.15 ± 0.62) × 10^–2^
(3.11 ± 0.04) × 10^4^	(1.10 ± 0.02) × 10^3^	5.80 ± 0.09	(1.19 ± 0.29) × 10^12^	9.1 ± 1.1	4.48 ± 0.35	4.27 ± 0.35	(0.90 ± 0.49) × 10^–2^
(3.11 ± 0.03) × 10^4^	(1.11 ± 0.02) × 10^3^	6.41 ± 0.29	0	0	5.35 ± 0.22	5.06 ± 0.20	(0.68 ± 0.25) × 10^–2^

### 2.3 Aerosol Size, Morphology, and Chemical Composition

The size, morphology, and chemical composition of the fresh and aged PS NPPs were analyzed using a SEMS, an Atomic Force Microscope (AFM, Bruker Dimension ICON AFM), and an HR-ToF-AMS, respectively. Fresh and aged PS NPPs were collected onto silica wafers for each aging condition, and their morphology was analyzed using an AFM, with details described in Supporting Information Section S2.2. Niu et al.^62^ established that the fragmentation ions C_6_H_6_^+^ and C_8_H_8_^+^, as detected by an HR-ToF-AMS, can be used as tracer ions for PS NPPs. To assess oxidation-induced chemical changes of PS NPPs, concentrations of C_6_H_6_^+^ and C_8_H_8_^+^ were monitored using the HR-ToF-AMS, as shown in Supporting Information Section S2.3.

### 2.4 Cell Culture and Exposure

#### 2.4.1 Collagen Coating

Low-height Millicell 6-well inserts were collagen-coated with a 1:4 solution of rat tail collagen and ethanol. The final concentration of the collagen solution was 1 mg/mL of rat tail collagen. The coated inserts were incubated for 2 h at room temperature before the collagen solution was aspirated. The inserts were dried overnight before cells were seeded the following morning.

#### 2.4.2 Cell Culture Materials

Minimum Essential Medium (MEM) Eagle Media, Millicell-CM Low Height culture plate inserts (PICMORG50), phosphate-buffered saline (PBS), and penicillin–streptomycin solution were purchased from Sigma-Aldrich. Trypsin (1:250), fetal bovine serum (FBS), and GlutaMAX (100×) were purchased from Gibco ThermoFisher Scientific.

#### 2.4.3 Cell Culture

The human bronchial epithelial cell line 16HBE14-0 (SCC150, Sigma-Aldrich Inc.) was cultured in MEM eagle medium supplemented with 5% FBS, 1% GlutaMAX (100X), and 1% penicillin–streptomycin at 37 °C and 5% CO_2_. Passages 4–22 were used for exposure experiments. This study utilized a commercially available human cell line. The cell line was not collected specifically for this research and does not contain identifiable private information. Therefore, institutional review board (IRB) or ethics committee approval was not required. All procedures were conducted in accordance with institutional biosafety and ethical guidelines for the use of established cell lines.

As shown in Figure 2, on Day 0, 1 mL of growth medium was added to the basolateral compartment of the inset before seeding to prewet the insets. After wetting the insets, 16HBE cells were seeded on the apical surface of collagen-coated low-height Millicell 6-well inserts at a seeding density of approximately 80,833 cells/cm^2^. Media changes were performed on Days 2 and 3 using warmed PBS to rinse the compartments before the addition of new growth media. On Day 4, the cells were brought to ALI. The culture medium was completely removed from the apical compartment, and the compartment was rinsed with PBS. The growth medium in the basolateral compartment was replaced with an equivalent volume of basal culture medium to supplement the cells from below. On Day 5, cells were exposed.

**Figure 2:**
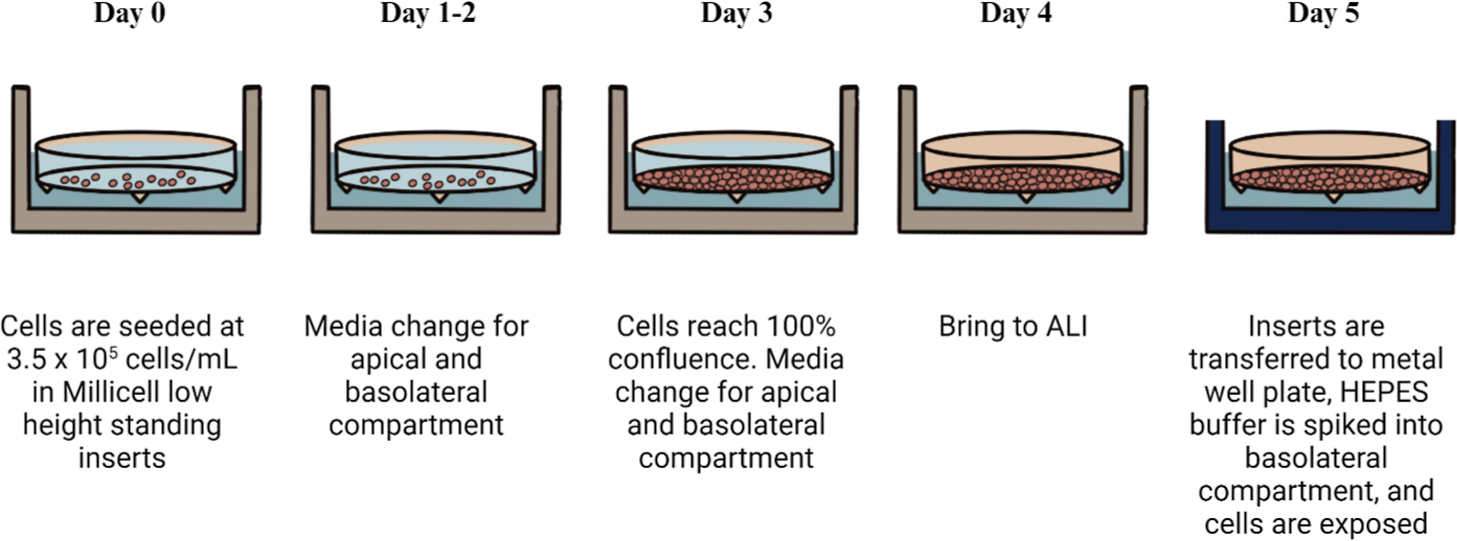
A schematic diagram to show the workflow of the cell culture for the air liquid interface (ALI) model. The human bronchial epithelial cell line (16HBE) was cultured in vitro at ALI before acute exposure to either filtered air or oxidized PS NPPs.

#### 2.4.4 Exposure

On Day 5, inserts were transferred into metal carrier 6-well plates. Immediately before exposure, 2-[4-(2-hydroxyethyl)­piperazin-1-yl]­ethanesulfonic acid (HEPES) buffer was spiked into the basolateral compartment for a target concentration of 20 mM HEPES. Cells in the metal carrier plates were placed into a CelTox with electrostatic precipitation features for PS-NPP exposure. A separate CelTox was used to expose cells to zero-grade air (control). Once placed into the respective CelTox sampler, the cells were simultaneously exposed to either a PS aerosol or zero-grade air for 3 h. After 3 h, the cells were moved from the CelTox sampler to an incubator for an additional 6 or 48 h before toxicity assessment.

### 2.5 Cytotoxicity

The ab102526 lactate dehydrogenase assay kit (colorimetric) was used to assess cell viability. The cell membrane releases lactate dehydrogenase (LDH) into the culture media upon injury. Following the assay protocol, 10 μL of sample media was added to a 96-well plate in duplicate. The wells were filled to a total volume of 100 μL by the subsequent addition of 40 μL of LDH assay buffer and 50 μL of reaction mix. The kit provided an internal positive control as well as NADH standards, which were added to the 96-well plate layout for each biological replicate. Once the reaction mix was added to each sample, the absorbance was measured immediately at OD 450 nm for 45 min every 3 min. LDH activity in the test samples was calculated as shown in eq 6.

6 LDHActivity=(BΔT×V)×D

here, *B* is the amount of NADH in the sample calculated from the standard curve, Δ*T* is the reaction time, *V* is the original sample volume added into the reaction well, and *D* is the sample dilution factor. The samples were not diluted, so the sample dilution factor was 1.

### 2.6 Gene Expression Analysis

#### 2.6.1 Lysis

Immediately after exposure, TRIzol was added to each 6-well insert to lyse the cells. The cells were incubated at room temperature for 2–3 min before being gently scraped. The cell lysate was collected in 1.5 mL Eppendorf tubes and stored at −80 °C until RNA and protein isolation.

#### 2.6.2 RNA Isolation

Cell lysate was thawed on ice before 1,3-bromobenzene was added for every 1.125 mL of TRIzol used for lysis. Samples were centrifuged to separate the aqueous phase (which contains the RNA) from the protein and DNA layers. The colorless aqueous phase was collected and stored in a separately labeled tube. Isopropanol was then added to the samples, which were incubated on ice for 10 min before additional centrifugation. The supernatant was discarded, and the RNA pellet was resuspended in RNase-free water.

#### 2.6.3 cDNA Synthesis and TaqMan qPCR

The QuantiTect Reverse Transcription Kit was used to remove genomic DNA from the samples and convert mRNA to cDNA for real-time qPCR. Sample cDNA was used immediately for PCR or stored at −20 °C. The synthesized cDNA was amplified with iTaq universal probes supermix (BIO-RAD) to perform real-time quantitative PCR (RT-qPCR) using a Roche LightCycler96 instrument. The primers used are listed in Table S1 of the Supporting Information. Gene transcription levels were analyzed using 2^–ΔΔCT^ method. β-actin (ACTβ) was used as the reference gene.

### 2.7 Protein Expression Analysis

A Bio-Plex Pro human chemokine immunoassay was used to analyze protein expression of IL-8, IL-6, TNF-α, and IL-1β. Following the kit’s provided protocol, 50 μL of antibody-coupled magnetic beads were added to each well of a 96-well layout. Samples, standards, and blanks were then added in duplicate to the 96-well plate at 50 μL per well. Cell culture media samples were not diluted for this assay. The plate was then covered with sealing tape and incubated on a shaker at 850 rpm for 1 h at room temperature. After incubation, the plate was washed to remove excess material that did not bind to the coupled beads. Detection antibodies were then added to each well, and the plate was recovered before being incubated on a shaker at 850 rpm for 30 min at room temperature. After incubation, 50 μL of 1X streptavidin-phycoerythrin (SA-PE) was added to each well, and the covered plate was placed on a shaker and incubated at 850 rpm for 10 min at room temperature. The plate was then washed three times with wash buffer before being placed on the shaker one final time to resuspend the beads before placing the plate on the reader for analysis. Bio-Plex Manager software was used for data acquisition and analysis. Detection parameters are shown in Table S2 of the Supporting Information. For Western blotting, cellular protein was extracted from the TRIzol lysate and stored in the −80 °C until total protein was quantified by BCA assay (ThermoFisher, #23225). 5× Laemmli loading buffer was added to the protein until a final concentration of 1× was reached. The protein was then heated at 95 °C for 10 min. For each sample, equal amounts of total protein were loaded into 10% SDS-PAGE gels, electrophoresed, and electrotransferred (125 V for 60 min or until the dye had reached the end of the gel) to 0.45 μm nitrocellulose membranes (Bio-Rad, #1620115) via wet transfer. Membranes were blocked at room temperature for 1 h using 5% BSA in 1× TBST and were incubated in a diluted primary antibody (Cell Signaling Technologies, #5853S) solution in blocking buffer overnight at 4 °C while rocking. Following primary antibody binding, membranes were washed three times for 5 min in 1X TBST. Membranes were then incubated in a 1:1000 diluted secondary antibody solution for 1 h at room temperature while rocking. Before imaging, membranes were rinsed with TBST three times for 5 min and then incubated with ECL solution (ThermoFisher, #34096) for 15 s.

### 2.8 Statistical Analysis

Statistical analyses compared LDH release, gene expression, and protein distributions between cells exposed to PS NPPs and the control (zero-grade air). Fold change in expression was calculated as the ratio of the average of three technical replicates for each treatment over a matched control. Then, a one-way Analysis of Variance (ANOVA) was used to compare the mean fold change of all treatment groups for each biological end point. There were three biological replicates for each end point (*n* = 3). All statistical analysis was conducted using GraphPad PRISM (Version 10.4.1) software.

## 3 Results

### 3.1 Equivalent Days of Aging

The water vapor content inside the PAM-OFR was 1.7 ± 0.2% throughout the experiments, and the O_3_ concentration varied from 4.46 ± 0.20 to 6.41 ± 0.29 ppm. The ^•^OH exposure derived from the PAM chem model program ranged from 0–2.7 × 10^12^ ± 2.9 × 10^11^ molecules cm^–3^ s^–1^. The ^•^OH exposures corresponded to 0, 9.1 ± 1.1, 16.5 ± 2.3, 20.9 ± 1.1 days of atmospheric aging, with parameters for each experiment summarized in Table 1. The simulated aging periods reflect environmentally relevant conditions as PS NPPs in the 300–900 nm range can persist in the atmosphere for weeks to months.^55^

### 3.2 Particle Size, Morphology, and Chemical Composition

The analyses of particle size, morphology, and composition in this study indicate changes in surface morphology of the particles during the selected range of atmospheric aging. Figure S2 from the Supporting Information shows the average size distributions for each of the experiments. Although there was a slight variation, the size distribution remained consistent between each oxidation condition. Trace nanoparticles (∼35 nm) were observed alongside the 500 nm PS NPPs, likely originating from impurities in the PS NPP solution or ultrapure water. However, their mass concentration was negligible relative to the 500 nm mode. Therefore, the measured cellular responses are expected to be dominated by the larger PS NPPs. The size distribution results were consistent with the atomic force microscopy images (AFM) seen in Figure S3 of the SI. Figure S3 demonstrates that the size remained consistent between unaged and maximum-aged conditions. The particles remained ∼ 500 nm spheres and did not develop cracks or major shape changes with increased oxidation. However, particles aged for 20 days displayed enhanced surface roughness, suggesting surface oxidation processes.

The HR-ToF-AMS results obtained in this study, as shown in Figures 3 and S4 of the Supporting Information, demonstrate alterations in the chemical composition of PS NPPs after aging. As shown in Figure 3, there was a significant decrease in C_6_H_6_^+^ and C_8_H_8_^+^ concentrations after about 5 days of atmospheric aging. Similarly, as shown in Figure S4 of the Supporting Information, the O/C ratio increased with higher ^•^OH exposure. Such results demonstrate that alterations in the chemical composition of PS NPPs were evident. The combination of the changes in C_6_H_6_^+^ and C_8_H_8_^+^ concentrations and the changes in the O/C ratio corresponds with the gene expression results as discussed below.

**Figure 3:**
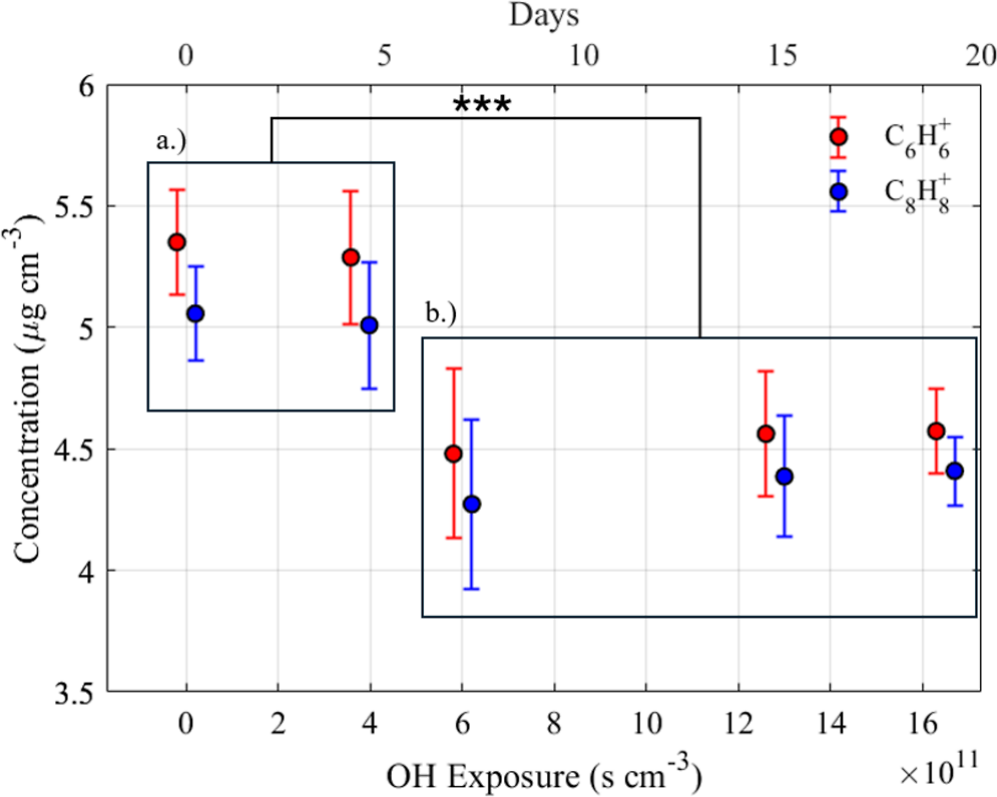
Average tracer ions (C_6_H_6_^+^ and C_8_H_8_^+^) concentrations as observed by the HR-ToF-AMS for various oxidation conditions. Note that all concentrations in box (a) are significantly higher than those in box (b) for both C_6_H_6_^+^ and C_8_H_8_^+^, demonstrating the mass loss of tracer ions due to aging. Unaged PS NPPs are 17.0% and 14.7% higher than max aged particles for C_6_H_6_^+^ and C_8_H_8_^+,^ respectively.

### 3.3 Cytotoxicity

Direct exposure to PS NPPs did not induce any significant changes in LDH activity for any of the time points or oxidation conditions, as shown in Figure S5.

### 3.4 Gene and Protein Expression

The qPCR results (Figure 4) showed significantly increased expression of *IL*-8 and *TNF*-α at both 6- and 48- hours. The highest aging condition (20.9 D) consistently induced *IL*-8 and *TNF*-α at both time points. HO-1 gene expression was also significantly increased in the higher aging conditions (16.5 and 20.9 days) but only at the 6 h time point.

**Figure 4:**
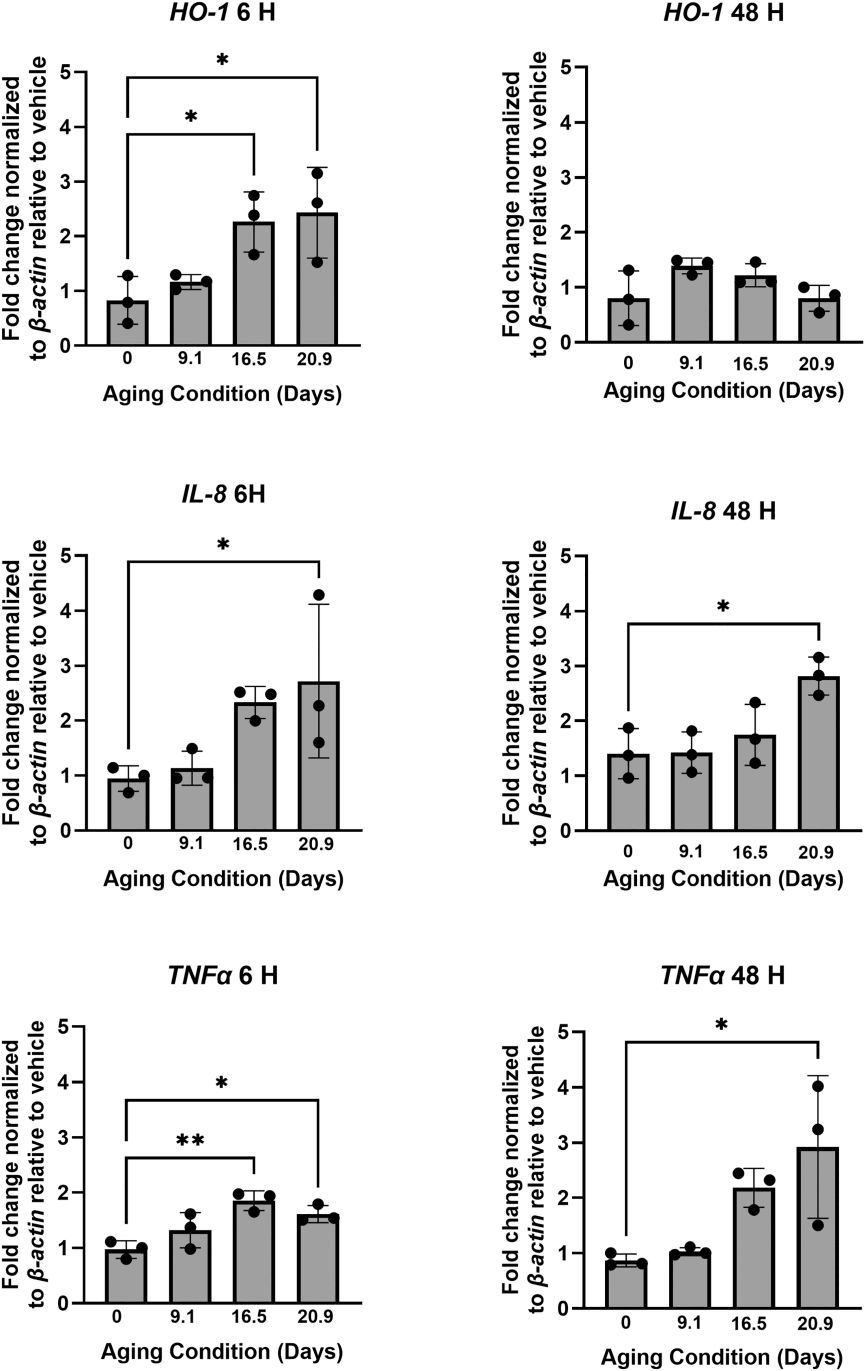
Results of gene expression analysis in 16HBE cells 6- and 48- hours postmicroplastics exposure. Error bars represent SD. Data analyzed using one-way ANOVA (*p < 0.05; **p < 0.01). Exact aging of samples from lowest to highest: 0, 9.1 ± 1.1, 16.5 ± 2.3, 20.9 ± 1.1.

IL-8, TNF-α, and IL-6 were analyzed at the protein level, in addition to IL-1β. IL-1β was not detected in any of the samples and therefore was not included. Protein expression (Figure 5) of IL-8 significantly increased at the 9.1 D aging condition after 6 h and at the 16.5 and 20.9 D aging conditions after 48 h. The latter end point matched gene expression data. Likewise, TNF-α was significantly increased at the maximum aging condition for both time points, mirroring gene expression data. IL-6 protein expression was also significantly induced at the maximum aging condition but only at the 48 h time point. Additionally, elevated expression of HO-1 was observed at the protein level at 48 h (Figure 6) following exposure to PS that had undergone the highest degree (20.9 days) of atmospheric aging.

**Figure 5:**
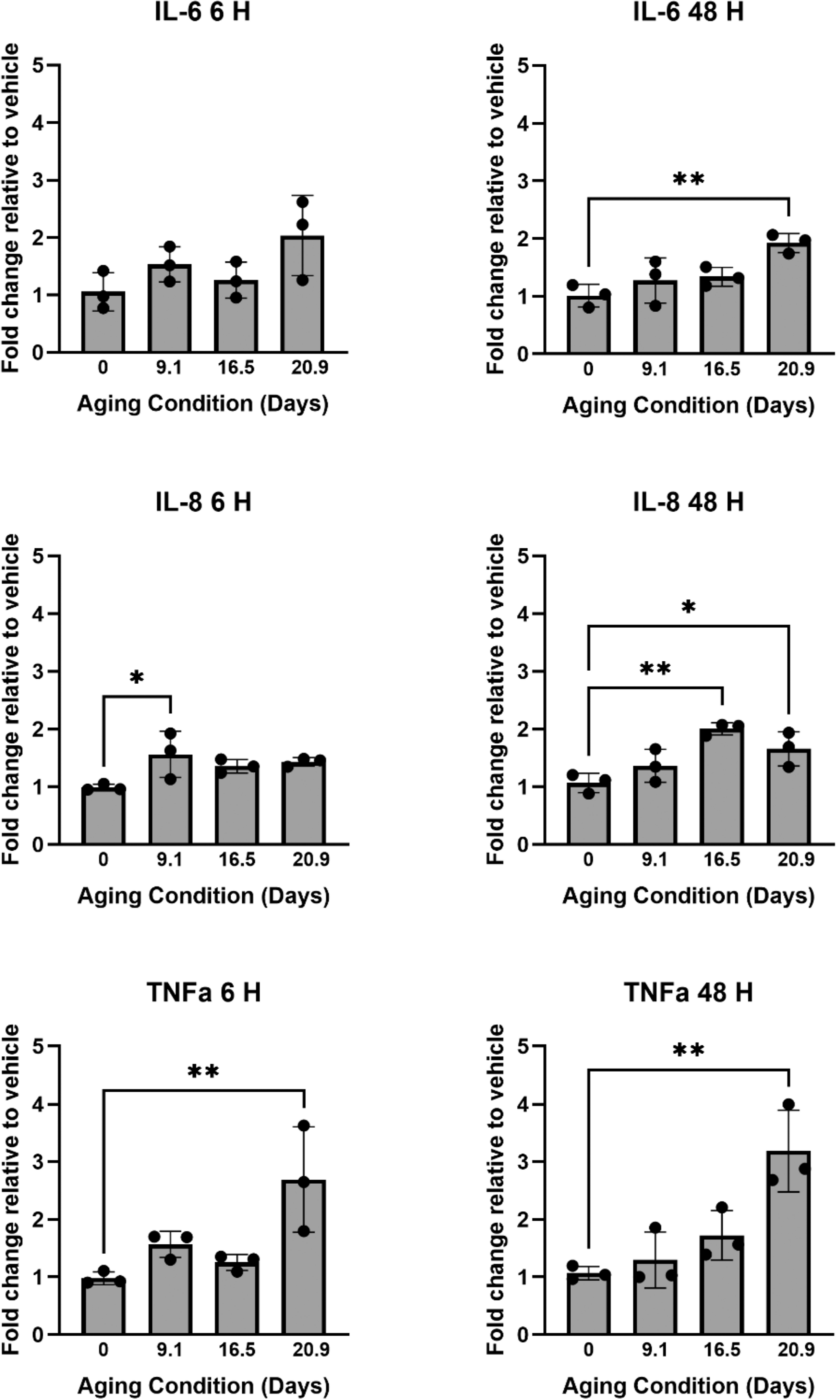
Results of protein expression analysis in 16HBE cells 6- and 48- hours post-microplastics exposure. Error bars represent Data analyzed using one-way ANOVA (*p < 0.05; **p < 0.01). Exact aging of samples from lowest to highest: 0, 9.1 ± 1.1, 16.5 ± 2.3, 20.9 ± 1.1.

**Figure 6:**
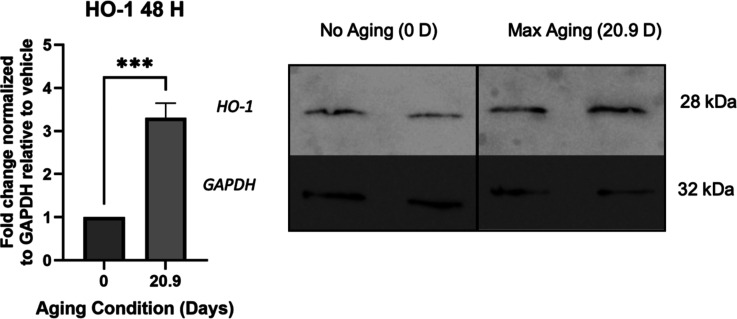
Protein expression of HO-1 following 48 H of exposure to aged polystyrene particles. Western blot of HO-1 and housekeeping gene GAPDH.

## 4 Discussions

### 4.1 Cytotoxicity, Inflammatory, and Oxidative Stress Responses in Bronchial Epithelial Cells

The PS particles in our study exhibited two modes centered around 35 and 500 nm. For these PS particles, we did not observe any enhancement in cytotoxicity, agreeing with the study by Goodman et al., where the A549 cell line was used, and the cell viability did not significantly decrease after exposure to PS-MP concentrations ranging from 10 to 100 μg/mL.^63^ PS particles in the latter study ranged in diameter from 1 to 10 μm. However, another study using PS-NPs with diameters of 25 or 70 nm observed increased cytotoxicity in A549 cells, a human lung epithelial cell line, starting at concentrations of 25 μg/mL for the smaller PS particles and 160 μg/mL for the larger particles.^64^ As such, it appears that after cells are exposed to similar concentrations of PS particles, particles with a larger diameter are less likely to affect lung cell viability. This could be due to the lower concentration employed (6.25 μg/mL) or a different cell type used. Prior studies also show that the inclusion of multiple cell types impacts cytotoxicity. For instance, one study exposed a monoculture of A549 cells and a coculture of A549 and THP-1 macrophages at ALI to 2 μg/cm^2^ PS-MPs for 28 days.^65^ There was no observable decrease in cell viability of the A549 monoculture, but the coculture experienced significant cell death at 28 days.

Gene and protein levels measured in our study revealed sustained effects on pro-inflammatory and oxidative stress markers in cells exposed at ALI to atmospherically aged PS NPPs. IL-8 expression increased in the PS NPPs aged 16.5 and 20.9 days. Numerous studies indicate IL-8 is a potent neutrophil attractor in the lung by binding to the chemokine receptors CXCR1 and CXCR2.^66^ Excessive production of IL-8 is linked to respiratory syndromes that exhibit a hyperactive inflammatory response, such as asthma and chronic obstructive pulmonary disease (COPD).^67^ Studies also show that a subset of patients with severe asthma have increased neutrophilic activity, which points to the importance of IL-8 signaling.^68,69^ PS-NPPs have been shown to induce IL-8 in A549 and BEAS-2B cells at higher concentrations than those tested in this study at similar time points.^52,64^ The results in this study indicate that aged polystyrene, even at lower concentrations, induces an inflammatory response after an acute respiratory exposure.

Additionally, TNF-α and IL-6 expression consistently increased in cells exposed to PS NPPs aged 20.9 days. TNF-α and IL-6 are pro-inflammatory cytokines associated with the pathogenesis of numerous diseases that are characterized by excessive inflammation. The upregulation of TNF-α activates immune cells and triggers the formation of different signaling complexes that have downstream effects of inducing cell death, inflammation, and host defense.^70^ An increase in TNF-α expression at both the mRNA and protein levels was observed at all time points at the maximum aging condition, indicating that the inflammatory potential of polystyrene increased after the MNPPs were oxidized for approximately 3 weeks.

In addition to assessing pro-inflammatory markers, it is useful to assess markers of oxidative stress. HO-1 acts as a cellular defender against oxidative stress in the lung. HO-1 acts as an antioxidant and anti-inflammatory agent by catalyzing the degradation of the oxidant heme into iron, carbon monoxide, and biliverdin.^71^ Therefore, an upregulation in HO-1 activity is considered a marker of oxidative stress, and increased HO-1 expression can be observed in acute respiratory distress syndrome (ARDS), COPD, cystic fibrosis, and other oxidant-induced lung injuries.^71^ An increase in HO-1 gene expression was detected in cells exposed to the aged PS NPPs (16.5 and 20.9 days) at the 6 h time point and appeared to resolve by 48 h. However, at the protein level, increased HO-1 expression was observed at 48 h in the highest aging conditions (20.9 days). Other studies have shown that exposure to unaged polystyrene can induce oxidative stress in lung cells at higher doses. Specifically, HO-1 activity and ROS formation have been shown to increase at 1000 μg/cm^2^ following a 20 min and 24 h exposure to unaged polystyrene. Our findings show collectively that at a lower dose (6.25 μg/mL), aged PS NPPs induce oxidative and pro-inflammatory markers in bronchial epithelial cells.

### 4.2 Particle Size, Morphology, and Chemical Composition

Based on this work, atmospherically oxidized PS NPPs induced stronger oxidative stress and inflammation compared than unaged particles, likely due to changes in chemical composition as well as physical changes. Decreased concentrations of tracer ions C_6_H_6_^+^ and C_8_H_8_^+^ (Figure 3), along with increased O/C ratios (Figure S4) with higher ^•^OH exposure, indicate that the enhanced cellular responses observed were driven by chemical changes to the surface of the PS NPPs. For instance, the gene and protein expression are significantly different after 16.5 or 20.9 days of aging compared to zero days of aging. This is consistent with the changes in both the tracer ion concentrations and O/C ratios. Previous studies show that the formation of hydroxy carbonyl functional groups is often observed with the oxidation of PS.^44^ Such chemical composition may impact the cell affinity of PS NPPs,^72^ thereby affecting their toxicity. Although there were no major detected changes to the size of the PS NPPs in this study, surface roughness was enhanced, agreeing with previous studies using larger, nonuniform particles, which observed cracks and changes upon aging.^45^ The results indicate that atmospheric aging could lead to both changes in surface morphology and composition of submicron particles.

We employed human bronchial epithelial cells cultured at ALI to characterize the differences in toxicity between unaged and aged polystyrene NPPs. To the authors’ knowledge, this is the first study investigating how atmospheric aging of NPPs affects human lung epithelium at the air–liquid interface. Atmospheric aging increased the pro-inflammatory responses to representative PS NPP inhalation exposure. This inflammatory response is possibly mediated through oxidative stress due to an observed upregulation of HO-1. These responses are likely due to changes in the chemical composition and surface morphology of the PS NPPs as the changes in C_6_H_6_^+^ and C_8_H_8_^+^ concentrations, O/C ratios, and surface roughness with increased aging corresponded to the cellular responses. Further research to evaluate other micro- and nanoplastics, such as nylon and polyethylene, following atmospheric aging would be useful to reflect the full diversity of realistic environmental exposures. These findings underscore the importance of incorporating environmental aging processes when evaluating microplastic and nanoplastic toxicity relevant to human inhalation exposure.

## Supplementary Material



## Notes

The Supporting Information includes Section S1: Polystyrene oxidation experimental set up, Section S1.1: Scanning electrical mobility spectrometer (SEMS) measurements, Figure S1: SEMS mass concentration of atomized polystyrene (PS) nanoplastic particle (NPP) solution, demonstrating the collection efficiency of the CelTox system, Section S1.2: Ozone measurements, Section S2: Particle size, morphology, and chemical composition, Figure S2: Average SEMS size distribution of atomized PS NPPs for each oxidation condition, Section S2.2: Atomic force microscope (AFM) imagery, Figure S3: Atomic force microscopy images of unaged and maximum aged PS NPPs, Section S2.3 High-resolution time-of-flight aerosol mass spectrometer (HR-ToF-AMS) measurements, Figure S4: Elemental ratios as a function of ^•^OH exposure as observed from the HR-ToF-AMS, Figure S5: Results of LDH assay after 6 and 48 h post microplastics exposure, Section S3: Gene expression, Section S3.1: qPCR, Table S1: Gene information for qPCR, Section S3.2: Enzyme-linked immunosorbent assay (ELISA), Table S2: Limits of detection for Bio-RAD human chemokine immunoassay (PDF)

## List of Abbreviations

AFM: atomic force microscope
ANOVA: analysis of variance
ALI: air liquid interface
ARDS: acute respiratory distress syndrome
COPD: chronic obstructive pulmonary disease
FBS: fetal bovine serum
16HBEs: human bronchial epithelial cell line
HEPES: 2-[4-(2-hydroxyethyl)­piperazin-1-yl]­ethanesulfonic acid
HO-1 (HO-1): heme oxygenase-1 protein (RNA)
HR-ToF-AMS: high-resolution time-of-flight aerosol mass spectrometer
IL-6 (*IL*-6): interleukin-6 protein (RNA)
IL-8 (*IL*-8): interleukin-8 protein (RNA)
MEM: minimum essential medium
NPP: nano-plastic particle
PBS: phosphate buffered saline
PAM: potential aerosol mass
PS: polystyrene
SEMS: scanning electrical mobility spectrometer
TNF-α (*TNF*-α): tumor necrosis factor protein (RNA)
