# Supplementary material for: Effects of Atmospheric Aging on the Respiratory Toxicity of Polystyrene Nanoplastic Particles
Source: Chem Res Toxicol. 2025 Nov 3;38(11):1905–15. doi: 10.1021/acs.chemrestox.5c00237 (PMC12956272; doi:10.1021/acs.chemrestox.5c00237)
Supplement: Supplementary file 1 [file tx5c00237_si_001.pdf]

Supporting Information

Effects of Atmospheric Aging on the Respiratory Toxicity of Polystyrene Nanoplastic Particles

Alana J. Dodero,[1ǂ] Olivia C. G. Lampe,[2ǂ] Sahir Gagan,[1] Sining Niu,[1] Natalie M. Johnson,[2,3*] Yue Zhang[1,3*]

[1] *Department of Atmospheric Sciences, Texas A&M University, College Station, TX, 77843 USA*

[3] *Department of Environmental and Occupational Health, Texas A&M University, College Station, TX, 77843 USA*

[2] *Interdisciplinary Faculty of Toxicology, Texas A&M University, College Station, Texas 77843, USA.*

October 2025

Chemical Research in Toxicology

ǂ*These authors contributed equally to the work.*

**Corresponding authors:* Natalie M. Johnson nmjohnson@tamu.edu,

Yue Zhang, yuezhang@tamu.edu

No. of pages: 8

No. of figures: 5

No. of tables: 2

S1. Polystyrene oxidation experimental set up

S1.1 Scanning electrical mobility spectrometer (SEMS) measurements

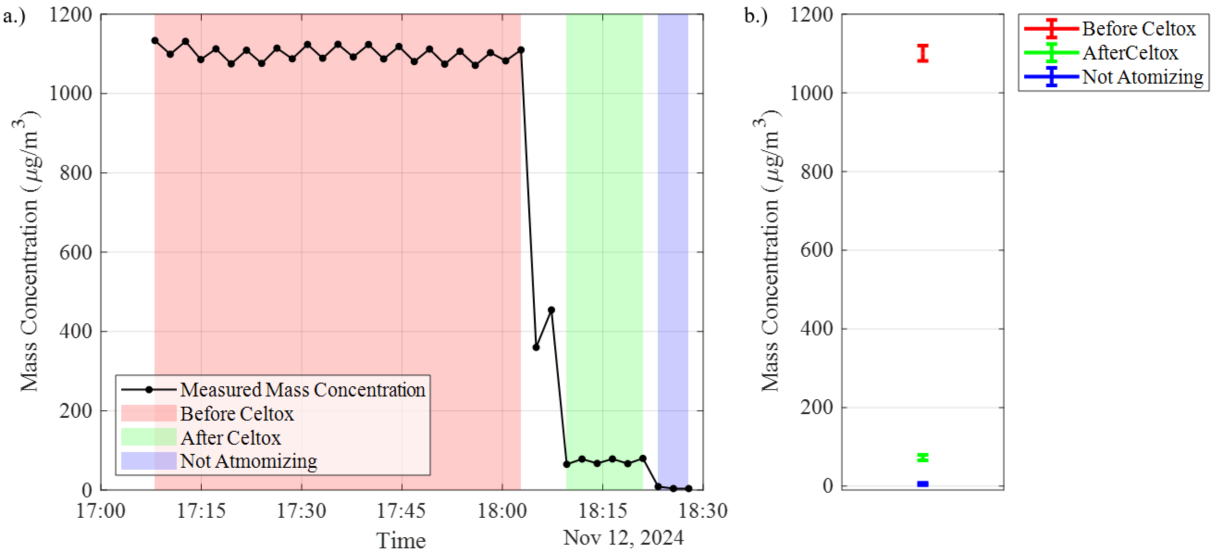

Figure S1. SEMS mass concentration of atomized polystyrene (PS) nano-plastic particle (NPP) solution, demonstrating the collection efficiency of the Celtox system. Figure S1a shows the SEMS mass concentration as a function of time before and after the Celtox system. Figure S1b shows the average concentrations before and after the Celtox system.

S1.2 Ozone measurements

During oxidation experiments, the ozone lamp voltage was kept constant, and any changes in ozone concentration were due to UV photolysis.

Control experiments were conducted to show that the O_3 denuder has a removal efficiency of 99.2%, quantified by an ozone monitor (2B technologies Model no. 202). The concentration before the O_3 denuder was 4690 ± 34 ppb, and the concentration after the denuder was 35 ± 14 ppb. Such removal efficiency reduces ozone concentration to below 50 ppb, ensuring that the cells were not exposed to excess O_3 from the PAM reactor.

S2. Particle size, morphology, and chemical composition

S2.1 SEMS size distribution

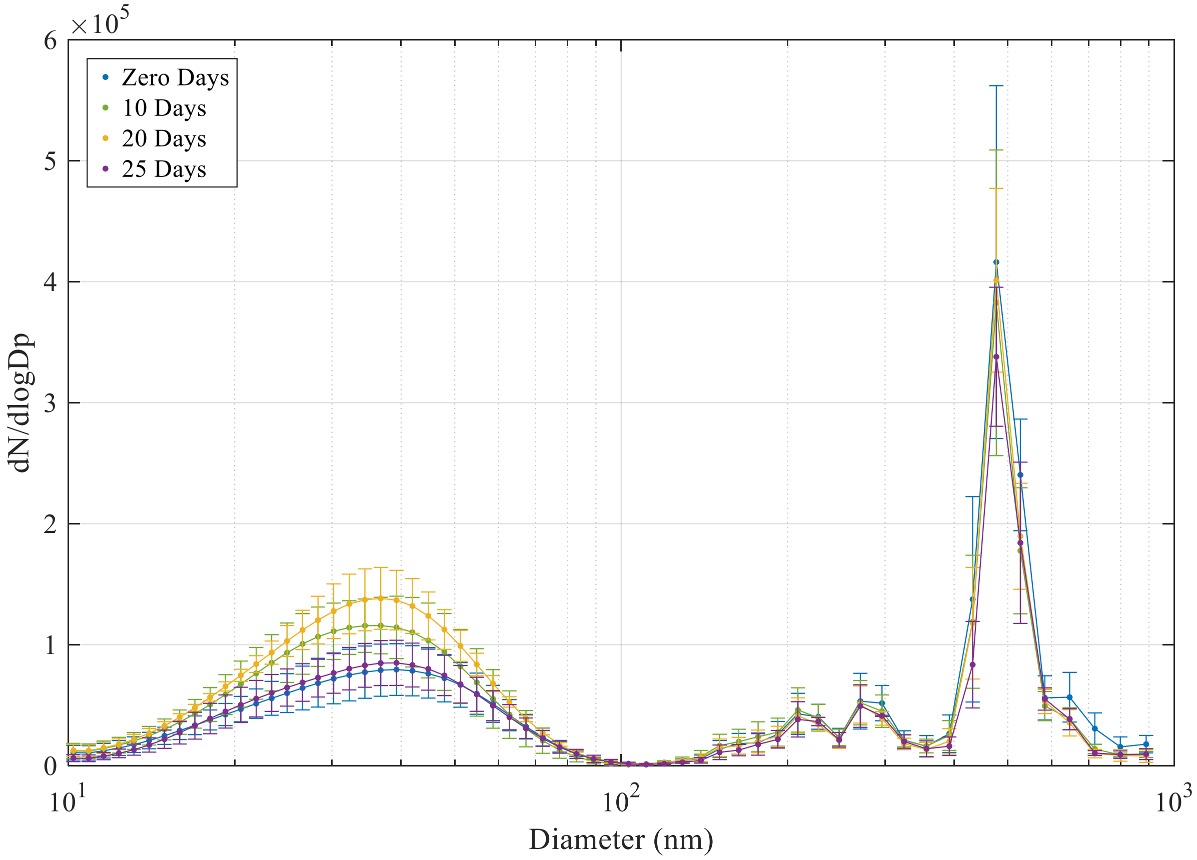

Figure S2. Average SEMS size distribution of atomized PS NPPs for each oxidation condition. The peaks at 500 nm and 35 nm represent PS NPPs and potential trace contaminants, respectively.

S2.2 Atomic force microscope (AFM) imagery

Aerosol particle samples were collected after each oxidation condition to analyze morphological changes induced by aging. Aerosols were impacted onto silica wafers (Silson Ltd) using a five-stage cascade impactor (Sioutas Cascade Impactor) with a flow rate of 9 lpm. Samples were stored at room temperature prior to analysis. They were imaged in a 2 x 2 µm grid using a Dimension ICON® AFM (Bruker) in tapping mode with a resonant frequency of 150 kHz and a spring constant of 5.4 N m^{-1}.

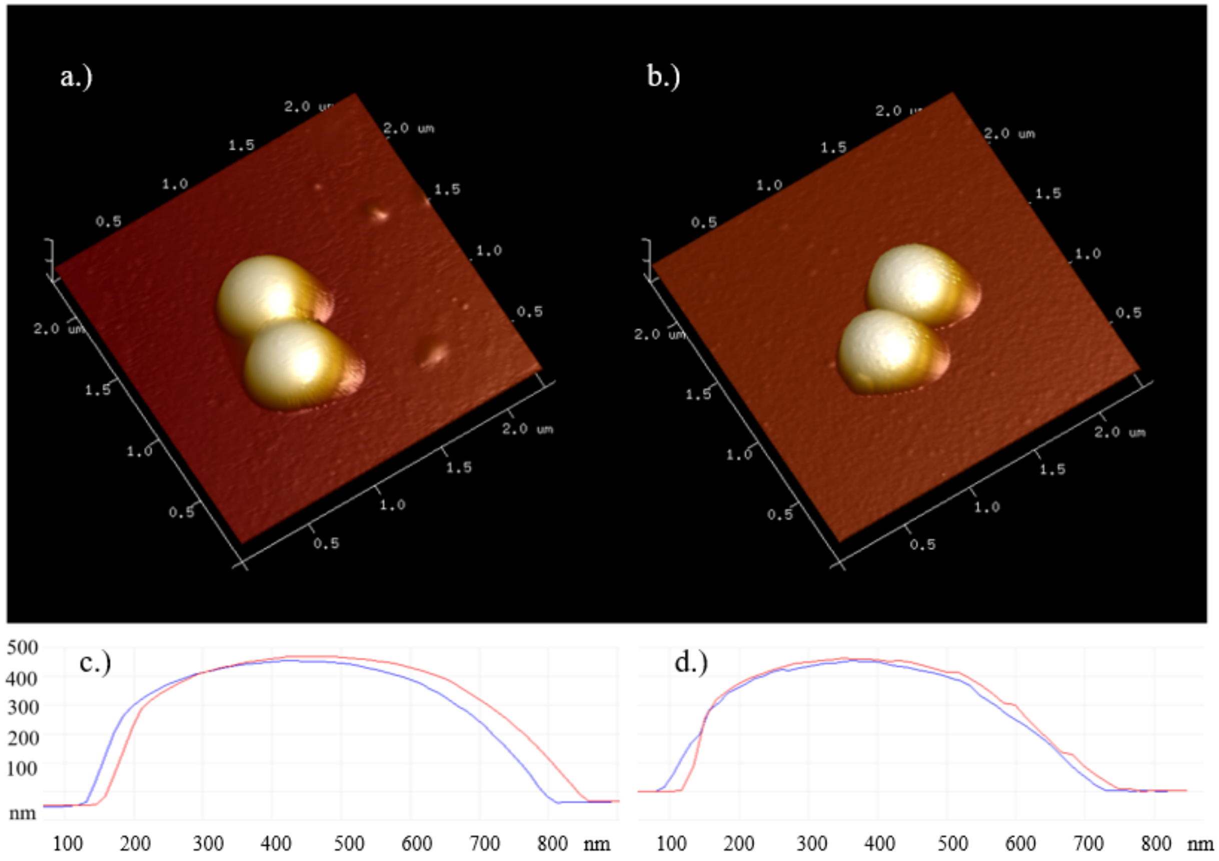

Figure S3. Atomic force microscopy images of unaged and maximum-aged PS NPPs. Figure S3a and S3b show 3-D plots of unaged and maximum-aged particles, respectively. Figure S3c and S3d represent two cross sections of the unaged and maximum-aged particles, respectively.

S2.3 High-resolution time-of-flight aerosol mass spectrometer (HR-ToF-AMS) measurements

The chemical compositions of fresh and aged PS NPPs were analyzed using an HR-ToF-AMS. The working principles of the HR-ToF-AMS are described previously.[1-3] The aerosol mass spectra were collected in V mode, and the vaporizer temperature was set to 600 °C.[3] The data were analyzed with the Squirrel (version 1.66) and Pika (version 1.26) packages in Igor Pro (WaveMetrics Inc., version 9).[4] The mass concentrations of tracer ions $C_6H_6^+$(m/z 78) and $C_8H_8^+$(m/z 104)[5] and elemental ratios between each oxidation condition were monitored to

determine the changes in chemical composition of PS NPPs induced by aging. While other techniques such as py-GC-MS, Raman spectroscopy, and FTIR have been successfully applied for offline detection of NPPs with high sensitivity,[6-8] the HR-ToF-AMS was selected here for its ability to provide real-time, online measurements of aerosol chemical composition under varying ·OH exposures. Previous studies have demonstrated the utility of the HR-ToF-AMS for analyzing NPPs.[5, 7, 9, 10]

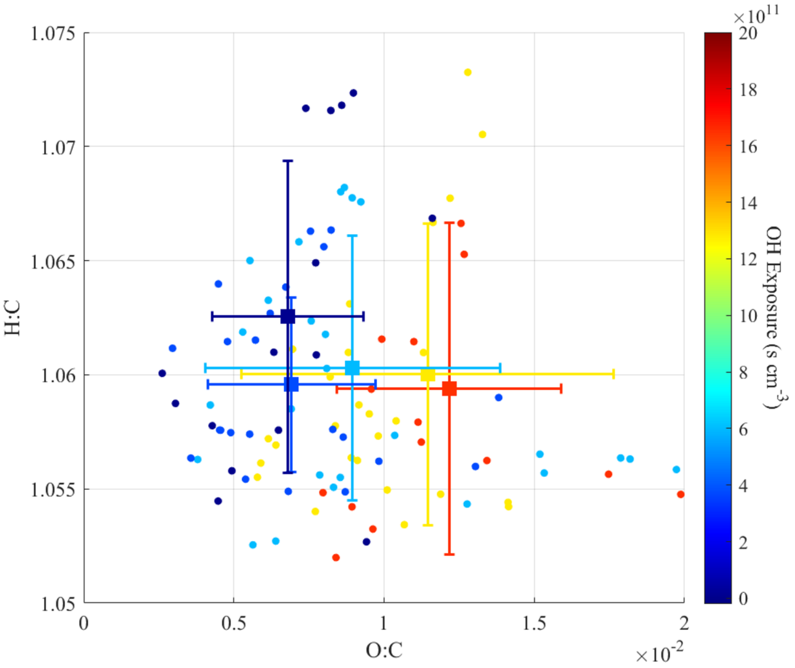

Figure S4. Elemental ratios as a function of ·OH exposure as observed from the HR-ToF-AMS. The circles represent the O:C and H:C ratios for each oxidation step, and the squares represent the average and standard deviation for each oxidation experiment. The color of the data points is determined by the ·OH exposure for each oxidation experiment.

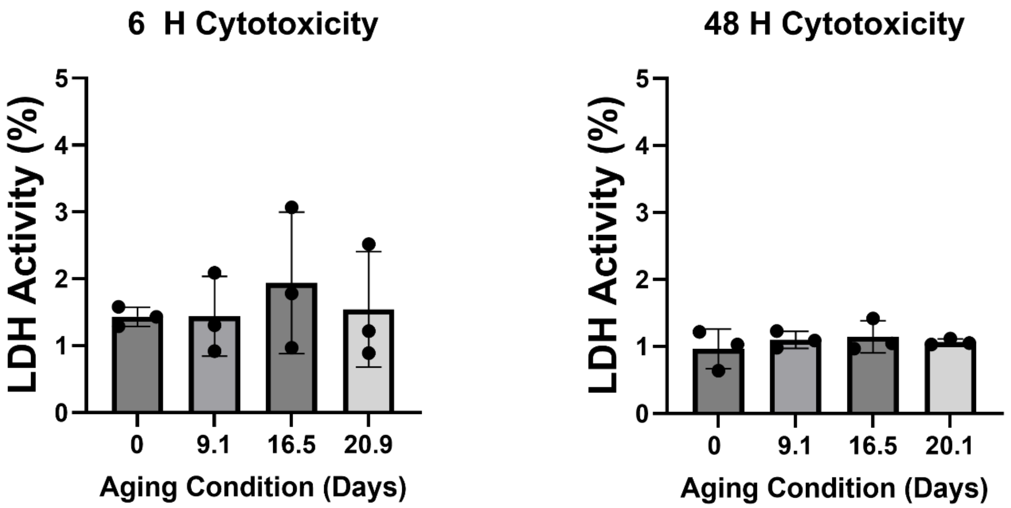

Figure S5. Results of LDH assay after 6- and 48-hours post-exposure to nanoplastics. Cytotoxicity was not observed at the tested doses. Exact aging of samples from lowest to highest: 0, 9.1 ± 1.1, 16.5 ± 2.3, 20.9 ± 1.1.

S3. Gene expression

S3.1 qPCR

Table S1. Gene information for qPCR. Primers and probe sequences for each target gene.

Gene	Forward Primer	Reverse Primer	Probe
ACTB	CTGGCACCCAGCACAATG	GCCGATCCACACGGAGTACT	ATCAAGATCATTGCTCCTCCTGAGCGC
HO-1	GAGGGTGATAGAAGAGGCCAAGA	GGTCAGCAGCTCCTGCAACT	TGCGTTCCTGCTCAACATCCAGCTC
IL-8	TTGGCAGCCTTCCTGATTTC	TATGCACTGACATCTAAGTTCTTTAGC	TGTGTGAAGGTGCAGTTTTGCCAAGG
TNF-α	CATCTACTCCCAGGTCCTCTT	TTGACCTTGGTCTGGTAGGA	ATGTGCTCCTCACCCACACCATC

S3.2 Enzyme-linked immunosorbent assay (ELISA)

Table S2. Limits of detection for Bio-RAD human chemokine immunoassay

Analyte	Bead Region	Undiluted Standard Value (pg/mL)	Working Range (pg/mL)	Limit of Detection (pg/mL)
IL-6	19	10,314	0.7-12,000	0.1
IL-8	54	3,942	0.5-7,640	0.04
IL-1β	21	1,693	0.4-7,000	0.1
TNF-α	36	21,984	0.9-13,879	0.2

References

(1) DeCarlo, P. F.; Kimmel, J. R.; Trimborn, A.; Northway, M. J.; Jayne, J. T.; Aiken, A. C.; Gonin, M.; Fuhrer, K.; Horvath, T.; Docherty, K. S. Field-deployable, high-resolution, time-of-flight aerosol mass spectrometer. *Analytical chemistry* **2006**, *78* (24), 8281-8289.

(2) Jayne, J. T.; Leard, D. C.; Zhang, X.; Davidovits, P.; Smith, K. A.; Kolb, C. E.; Worsnop, D. R. Development of an Aerosol Mass Spectrometer for Size and Composition Analysis of Submicron Particles. *Aerosol Science and Technology* **2000**, *33* (1-2), 49-70. DOI: 10.1080/027868200410840.

(3) Jimenez, J. L.; Jayne, J. T.; Shi, Q.; Kolb, C. E.; Worsnop, D. R.; Yourshaw, I.; Seinfeld, J. H.; Flagan, R. C.; Zhang, X.; Smith, K. A. Ambient aerosol sampling using the aerodyne aerosol mass spectrometer. *Journal of Geophysical Research: Atmospheres* **2003**, *108* (D7).

(4) Stark, H.; Yatavelli, R. L. N.; Thompson, S. L.; Kimmel, J. R.; Cubison, M. J.; Chhabra, P. S.; Canagaratna, M. R.; Jayne, J. T.; Worsnop, D. R.; Jimenez, J. L. Methods to extract molecular and bulk chemical information from series of complex mass spectra with limited mass resolution. *International Journal of Mass Spectrometry* **2015**, *389*, 26-38. DOI: https://doi.org/10.1016/j.ijms.2015.08.011.

(5) Niu, S.; Liu, R.; Zhao, Q.; Gagan, S.; Dodero, A.; Ying, Q.; Ma, X.; Cheng, Z.; China, S.; Canagaratna, M.; et al. Quantifying the Chemical Composition and Real-Time Mass Loading of Nanoplastic Particles in the Atmosphere Using Aerosol Mass Spectrometry. *Environmental Science & Technology* **2024**, *58* (7), 3363-3374. DOI: 10.1021/acs.est.3c10286.

(6) Shi, Y.; Shi, L.; Huang, H.; Ye, K.; Yang, L.; Wang, Z.; Sun, Y.; Li, D.; Shi, Y.; Xiao, L.; et al. Analysis of aged microplastics: a review. *Environmental Chemistry Letters* **2024**, *22* (4), 1861-1888. DOI: 10.1007/s10311-024-01731-5.

(7) Tawadrous, M. A. R.; Lee, A. K. Y.; Chan, A. W. H. Characterization of airborne PET nanoplastic particles using Aerosol mass spectrometry. *Aerosol Science and Technology* **2025**, *59* (6), 691-704. DOI: 10.1080/02786826.2025.2451070.

(8) Xu, J.-L.; Thomas, K. V.; Luo, Z.; Gowen, A. A. FTIR and Raman imaging for microplastics analysis: State of the art, challenges and prospects. *TrAC Trends in Analytical Chemistry* **2019**, *119*, 115629. DOI: https://doi.org/10.1016/j.trac.2019.115629.

(9) Gagan, S. D., Alana; Liu, Ruizhe; Cheng, Zezhen; Niu, Sining; Kim, Yeaseul; Lambe, Andrew; Chen, Yuzhi; China, Swarup; Zhang, Yue. Characterizing the Kinetics of Chemical Aging and Cloud Condensation Nuclei Activity of the Atmospheric Polystyrene (PS) Nanoplastic Particles (NPPs). *Environmental Science & Technology* **2025**.

(10) Petters, S. S.; Kjærgaard, E. R.; Hasager, F.; Massling, A.; Glasius, M.; Bilde, M. Morphology and hygroscopicity of nanoplastics in sea spray. *Physical Chemistry Chemical Physics* **2023**, *25* (47), 32430-32442, 10.1039/D3CP03793B. DOI: 10.1039/D3CP03793B.
